# Chitosan-insulin nano-formulations as critical modulators of inflammatory cytokines and Nrf-2 pathway to accelerate burn wound healing

**DOI:** 10.1186/s11671-023-03941-2

**Published:** 2023-12-12

**Authors:** Deepinder Sharda, Sandip Ghosh, Pawandeep Kaur, Biswarup Basu, Diptiman Choudhury

**Affiliations:** 1 Department of Chemistry and Biochemistry, Thapar Institute of Engineering and Technology, Patiala, Punjab 147004 India; 2 Department of Neuroendocrinology and Experimental Hematology, Chittaranjan National Cancer Institute, Kolkata, 700026 India; 3 Centre of Excellence for Emerging Materials, Thapar Institute of Engineering and Technology, Patiala, Punjab 147004 India

**Keywords:** Insulin, Chitosan, Burn wound healing, Inflammatory cytokines, Nrf-2 pathway

## Abstract

Burn injuries are characterized by prolonged inflammatory phases, neurovascular damage, and hypermetabolism, eventually causing improper tissue regeneration. Insulin has gained considerable attention in normal and diabetic wound healing, yet its role in burn wounds remains poorly understood. In this study, insulin-chitosan nano-formulations (ICNP) were synthesized using a simple and robust mechanism and characterized to monitor specific interactions between insulin and chitosan, and the particles measuring approximately 30 nm in size exhibited mild alterations in the amide I, II, and III bonds of the insulin protein along with impressive insulin loading efficiency of 88.725 ± 0.295% under physiological conditions, and significantly improved burn wound healing in vitro (HEKa cells) and in vivo (murine third-degree burn model). The underlying mechanism behind superior wound closure and tissue remodeling was attributed to significant early phase reduction of pro-inflammatory cytokine IL-6 levels in ICNP-treated mice, while anti-inflammatory cytokine IL-10 levels became markedly elevated, resulting in enhanced re-epithelialization and collagen deposition. Furthermore, treatment of ICNP was associated with unregulated expression of Nrf-2, a key regulator of oxidative stress and inflammation, indicating their molecular crosstalk. These findings highlight the potential of ICNP as a promising therapeutic formulation for burn wound healing, promoting wound closure by modulating inflammatory phases, making it a valuable candidate for further clinical development in burn care.

**Graphical Abstract:**

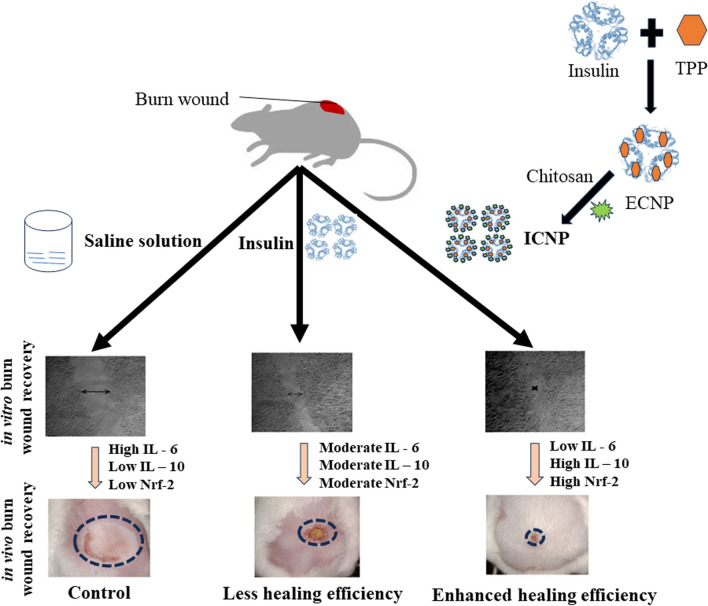

**Supplementary Information:**

The online version contains supplementary material available at 10.1186/s11671-023-03941-2.

## Introduction

Wound healing involves sequential phases, including inflammation, proliferation, and remodeling, to maintain tissue integrity [1, 2]. The cost of healing acute and chronic wounds ranges from $28.1 to $96.8 billion, with surgical wounds and diabetic ulcers incurring the highest expenses [3]. Burn injuries are particularly debilitating and painful, leading to increased morbidity and mortality rates. According to the World Health Organization (WHO), burn injuries cause 180,000 deaths annually, with approximately 11 million people requiring medical assistance globally as per report of 2018. The number of patients affected in India was around 1 million, whereas in the USA, the number is nearly half a million [4]. Thermal burns, which can result from dry (fire/flame) or wet (scald) sources, account for approximately 80% of all burns and are classified based on wound depth [5]. When a thermal injury covers over 20% of the body's surface area, it is called a burn shock [6].

Burn injuries are divided into three distinct categories: First-degree (damaged epidermis), Second-degree (damaged epidermis and part of dermis), and Third-degree (damage extended up to hypodermis) based on the severity of the injury [7]. Third-degree burns, histologically distinct from superficial burns, are characterized by destruction of the epidermis, dermis, and part of hypodermis, coagulative necrosis, absence of epidermal regeneration, and pronounced inflammatory response. These burns necessitate surgical intervention, such as skin grafts, due to their inability to self-regenerate. Histological analysis may reveal granulation tissue formation in later stages, often leading to hypertrophic or keloid scarring. Wound healing involves a balance between pro-inflammatory and anti-inflammatory pathways, but burn injuries disrupt this process [8]. Burn injuries are distinct from other wounds in some respects, such as the degree of systemic inflammation [9], electrolyte and fluid loss, compromised or necrotized wound edges, and tendency towards shock or coma [10]. They are characterized by enhanced capillary permeableness, greater systemic vascular resistance, reduced cardiac output, increased hydrostatic pressure across microvasculature, and movement of proteins and fluids to the interstitial space from intravascular space [11]. The altered immune system increases the susceptibility to infection, eventually leading to sepsis and thus aggravating systemic inflammation [12]. Microbial infection arising from multi-drug resistant bacteria and fungi is becoming the primary cause of mortality in patients with burn wounds [13, 14]. Further, the extent and depth of the burn are directly proportional to the extent of hypermetabolism and inflammation, which are responsible for impaired wound healing by delaying re-epithelialization [15, 16]. These changes in the pathophysiology have a more significant impact on the pharmacodynamics and pharmacokinetics of the drugs used for healing burn wounds. The major challenge before the researchers is burn management due to its slow healing rate, pain, higher infection susceptibility, and hypertrophic scarring [17]. Clinical practices, including skin grafting, skin substitutes, wound dressings, and negative pressure wound therapy, are being explored for burn healing. Apart from them, various other treatments are under consideration for the treatment of burn pain (Ketamine, sedatives, anxiolytics, pruritus, and neuropathic drugs) and scar management (mesenchymal stem cells and adipose-derived stem cells) along with pharmacological approaches (silicone, corticosteroids, transforming growth factor-β modulators) and surgical approaches (fat grafting, laser therapy for scars, ablative and non-ablative fractional lasers) [4, 18–20]. Although these methods have huge potential, they have limited usefulness as they primarily focus on wound closure rather than addressing the underlying pathophysiology [18]. Limitations include delayed healing, low mechanical strength, and reduced aeration [8]. These factors lead to prolonged recovery, increased recurrence rates, treatment failure, amputations, and higher costs for patients [18, 21–23].

Keratinocytes play a crucial role by immediately reaching the wound site and starting their healing function, which further leads to alterations in gene expression profiles. They stimulate the growth of myofibroblast-like cells responsible for wound contraction and also secrete signaling molecules that act either in an autocrine or paracrine manner, causing the pleiotropic effect on various cell types, which in turn promote the activation of keratinocytes during wound closure by secreting signaling molecules [24, 25].

Achieving a balance between pro-inflammatory and anti-inflammatory cytokines is crucial for effective burn wound healing. Pro-inflammatory cytokines, such as tumor necrosis factor-alpha (TNF-α), interleukin-1 beta (IL-1β), and interleukin-6 (IL-6), are released in burn wounds, leading to tissue damage and delayed healing. Anti-inflammatory signaling pathways, like the Nrf-2 (Nuclear factor erythroid 2-related factor 2) pathway, counteract inflammation and promote tissue repair by increasing anti-inflammatory cytokines, like interleukin-10 (IL-10) that help to resolve inflammation and promote tissue repair [26, 27]. Nrf-2 is a cytosolic transcription factor that plays a crucial role in cellular defense by activating antioxidant and detoxifying enzymes [28, 29]. It helps prevent excessive reactive oxygen species (ROS) production in inflamed and wounded tissues, suppressing chronic inflammation. Nrf-2 expression is significant in keratinocytes and macrophages, promoting matrix and granulation tissue formation [30, 31]. It also activates the production of TGF-β (Transforming Growth Factor- beta) and protects cells from ROS-induced damage. The Nrf-2 antioxidant pathway is vital for cellular protection against inflammation and environmental stressors [32]. Bone marrow mesenchymal stem cells are found to promote burn wound healing by activating the Akt/mTOR (protein kinase B/mammalian Target of Rapamycin) pathway [33].

Recently, proteins and growth factors, including insulin, have become common and effective therapeutic choices for tissue regeneration and wound healing [34]. Insulin regulates various cytokines, growth factors, and hormones through selective mechanisms. It reduces inflammation by inactivating NF-kβp50/p65 (Nuclear factor kappa-light-chain- enhancer of activated B cells) and TNFα-mediated inflammatory pathways [34]. Insulin modulates protein synthesis, promoting cell growth and differentiation and enhancing cell survival by inhibiting proteolysis via FOXO (Forkhead Box O) inactivation [8]. Additionally, insulin promotes the secretion of anti-inflammatory cytokines and reduces the secretion of pro-inflammatory cytokines, thereby modulating wound inflammation [35]. Nanoparticle-based insulin delivery has shown enhanced selectivity, sensitivity, and effectiveness compared to free insulin [36–38]. These nanocarriers improve the therapeutic potential of the drug manifold by acting as site-specific delivery agents due to their functionalization with specific moieties, which add to their efficacy [39–41]. This has led to the development of various insulin-based and other antidiabetic protein-based drugs that either improve insulin sensitivity or stimulate increased insulin secretion from the pancreas to treat wounds under normal or diabetic conditions [42–45]. Insulin-loaded Poly-DL-lactide/glycolide nanoparticles were developed for topical delivery of insulin to treat skin burn wounds by regeneration of skin [46]. Other proteins like silk fibroin are found to be effective against burn wounds when delivered in combination with gelsevirine as a multilayer dressing [47].

Nanotherapeutics involving metals and polymers has enormous potential in treating burn wounds [48]. Different naturally occurring biopolymers have emerged as potential carriers for controlled drug delivery. One such biodegradable polymer is chitosan [49]. Chitosan is a cationic heteropolymer derived from chitin, which is commonly found in the exoskeleton of shrimp, fungi, and yeasts [50]. In wound healing, chitosan plays a crucial role by providing a matrix for three-dimensional tissue growth, stimulating tissue organization and cell proliferation. It also activates macrophages and enhances their tumoricidal activity. Additionally, chitosan assists in natural blood clotting and reduces pain at the wound site by blocking nerve endings [51]. It promotes hemostasis, keratinocyte proliferation, and improved adhesion. Furthermore, chitosan can be easily modified to be efficiently degraded by body enzymes, making it a promising candidate for burn wounds [52–54]. Chitosan nanoparticles are found to be preventive against collagen-induced severe arthritis in Wistar rats when loaded with zinc gluconate [55]. The hydrogel films were developed using carboxy methyl chitosan and hydroxyethyl cellulose for potent fibroblast growth factor-2 (FGF-2) delivery to the dermal tissue injury site [56]. Similarly, chitosan/alginate nanoparticles were used to heal burn wounds by delivering Esculentoside A at the burn site [57]. The present study aims to synthesize insulin-loaded chitosan nano-formulations and investigate human insulin's structural and functional changes after interaction with chitosan under various physiological conditions. The effectiveness and efficiency of these formulations will be evaluated using in vitro and in vivo models in terms of wound healing activity, with a focus on mechanistic aspects.

## Materials and methods

### Materials

The required materials for this study were sourced as follows: sodium triphosphate, chitosan, NaOH, and HCl were purchased from Loba Chemie, India, while recombinant human insulin was procured from Elli Lilly, India. DMEM (Dulbecco's Modified Eagle Medium) cell culture media, Fetal Bovine Serum (FBS), and penicillin–streptomycin were acquired from HiMedia, India. All other chemicals utilized in the study were of research grade and obtained from HiMedia.

### Synthesis of ECNP and ICNP nanoparticles

**Fig. 1:**
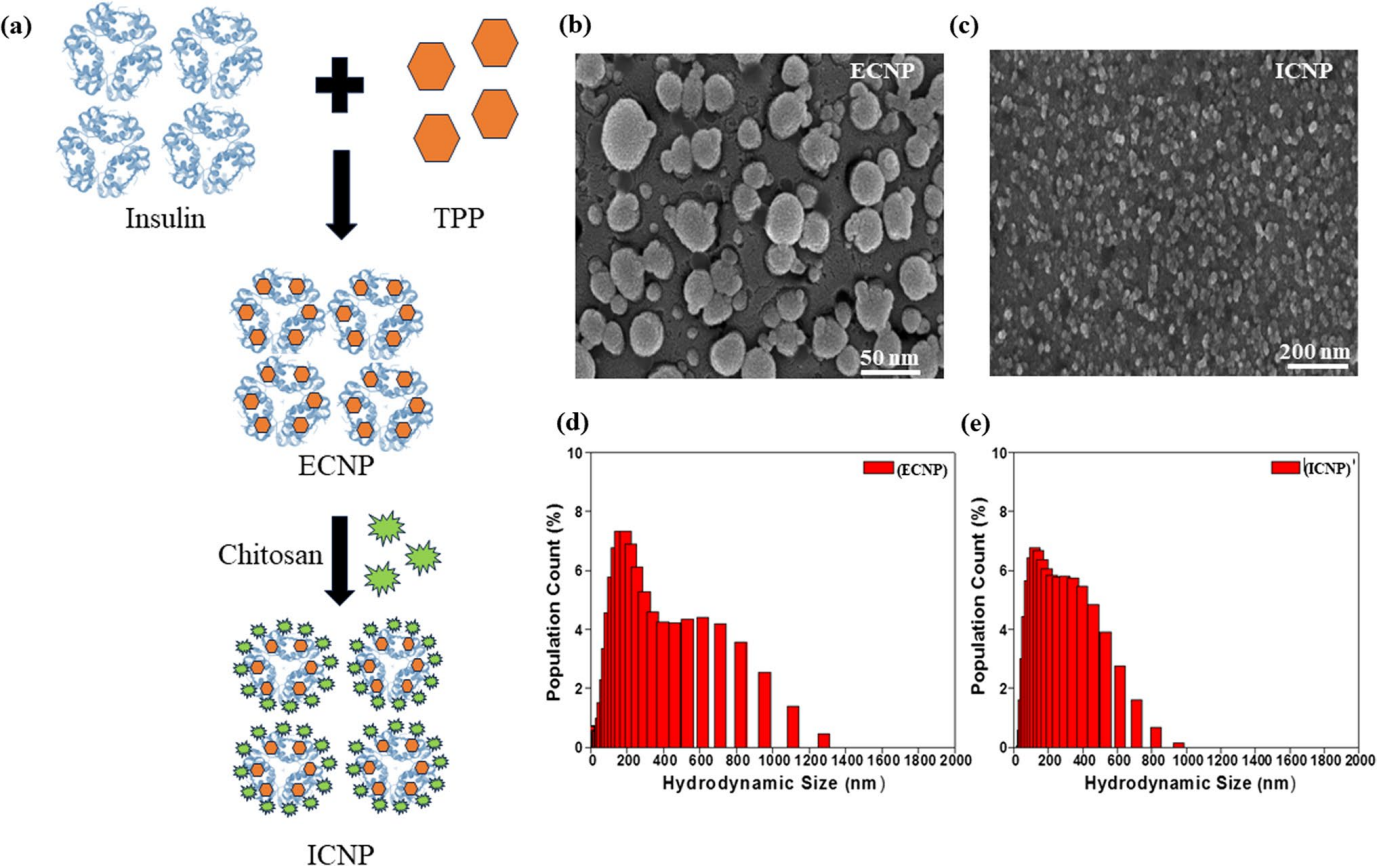
The figure shows the schematic synthesis and morphological studies of synthesized ICNPs **a** a brief synthesis procedure for making the ICNP nanoparticles. FE-SEM image of **b** ECNP particles and **c** ICNP nanoparticles at pH 9 indicating the formation of spherical particles of size 160 nm and 30 nm, respectively, at a scale of 500 nm and 200 nm. Dynamic light scattering for determining the hydrodynamic size of **d** ECNP particles and **e** ICNP particles at a pH of 9

The formation of TPP (Tripolyphosphate) mediated ECNP (empty chitosan-TPP nanoparticles) and ICNP (insulin-loaded chitosan-TPP nanoparticles) nanoparticles was achieved through a straightforward procedure. Initially, chitosan (10 mg) was dissolved in double distilled water, and the pH was adjusted to 6 by adding the acetic acid solution to facilitate the proper dissolution of chitosan. A solution of TPP previously dissolved in 4 ml of double distilled water was prepared separately and stirred for 10 min at room temperature. This TPP solution was then added dropwise to the chitosan solution, and the resulting mixture was stirred at room temperature for 1 h. The resulting solution was labeled as ECNP (empty chitosan-TPP nanoparticles) nanoparticles. To synthesize ICNP (insulin-loaded chitosan-TPP nanoparticles) nanoparticles, the TPP solution described above was mixed with 40 µl of insulin solution and stirred for 10 min at low rpm and room temperature. Subsequently, this solution was added dropwise to the chitosan solution and stirred for an hour at room temperature. Different solutions were prepared by adjusting the pH of the chitosan solution to obtain solutions with pH values of 6, 7.4, 8, and 9. (Fig. 1a).

### Particle size and morphological analysis

We have performed DLS (dynamic light scattering) to find out the average hydrodynamic size of ECNP and ICNP using a Malvern DLS-Zeta size analyzer. Also, the zeta potential was measured to check out the stability of the formed samples using the same instrument. After that, FE-SEM (Field Emission-Scanning Electron Microscopy) analysis was performed to determine the morphology of ICNP. For this, the samples were centrifuged at 240 rpm for nearly 10–15 min, followed by thoroughly washing the pellet to remove unbound salt or impurities associated with the sample.

### FTIR Analysis

To identify the functional group alterations in chitosan, TPP, ECNP, insulin, and ICNP, an Agilent Cary 600 series FTIR (Fourier-Transform Infrared Spectroscopy) Spectrophotometer was employed. Before analysis, the samples were washed to eliminate impurities and were subsequently dried at 37 °C. Next, the dried samples were mixed with potassium bromide (KBr) to create pellets, which were then subjected to scanning in the spectral range of 500 cm-1 to 4000 cm^−1^. This allowed for the comprehensive examination of the samples' infrared absorption characteristics and the determination of their respective functional groups [58].

### Protein loading and release kinetics

The chitosan nanoparticles were utilized for loading human insulin, and their drug-loading capacity and release kinetics were investigated. The particles were suspended in 30 ml of PBS (Phosphate Buffered Saline) buffer with a pH of 7.4 and the system was maintained at a temperature of 37 °C while subjecting it to a rotational speed ranging between 130 and 150 rpm. At regular time intervals, samples of 2 ml were collected from the suspension, and their absorbance was measured. A UV-2600 spectrophotometer (Shimadzu) equipped with a 4000 µl quartz cuvette with a path length of 1 cm was employed for the measurements, operating within 200–800 nm. The obtained absorbance values were then plotted against time to analyze the trend of drug release over time [59].

### Cell viability studies

To assess cell viability, the HEKa cell line [Human Epidermal Keratinocytes (adult)] was utilized, and an MTT [3-(4,5-dimethylthiazol-2-yl)-2,5-diphenyltetrazolium bromide] assay was conducted. HEKa cells were seeded in 96-well plates at a density of 1 × 104 cells per well and allowed to reach a confluency of 75–80%. Subsequently, the cells were incubated with four different concentrations (1.5, 7.5, 30, and 60 µM) of ECNP, insulin, and ICNP. The cells were then incubated at 37 °C for 24 h. After the incubation period, the media was removed, and an MTT solution (2 mg/ml in 5% ethanol) was added to each well, followed by further incubation. After 3 h, the media-MTT mixture was discarded, and 200 µl of dimethyl sulfoxide (DMSO) was added to dissolve the formazan crystals. The absorbance of the resulting solution was measured at 570 nm.

To calculate the inhibition percentage, the following equation was employed:

a $$\% {\text{ inhibition}} = \left[ {{1} - \left( {{\text{At}}/{\text{Ac}}} \right)} \right] \times {1}00$$

where At represents the absorbance of the test substance, and Ac represents the absorbance of the control solvent. The experiment was performed in triplicates for each concentration, and the average values were used to generate the corresponding graphs.

### in vitro* wound healing assay*

Human Epidermal Keratinocyte (HEKa) cells were employed for conducting the in vitro wound healing assay using the prepared nanoparticles. The cells were cultured in 35 mm plates using DMEM media supplemented with antibiotics. Subsequently, the cells were placed in an incubator at 37 °C with a 5% CO_2_ supply, providing an optimal environment for cell growth. Once the cells reached a 90–95% confluency, the scratch method was employed to create wounds. Different concentrations (1.5, 7.5, 30, and 60 µM) of ECNP, insulin, ICNP, and media were added to the cells and incubated. The wound diameters were measured at specific time intervals, including 6 h, 12 h, and 24 h, to assess the changes in diameter over time and evaluate the effects of the nanoparticles on the wound healing process.

### Determination of combination index (CI) for chitosan-insulin

The quantitative measurement of getting the combined effect of two individual drugs is called the Combination Index (CI). Whether the two different drugs will exhibit synergism or antagonism is determined by calculating the drug combination index. A synergistic effect is observed if the different drugs administered together act together to increase each other's activity and is given by a value of less than 1 (CI < 1). On the other side, if the CI value comes out to be more than 1 (CI > 1), the antagonistic effect is shown, indicating that the action of one drug is inhibited by another. And, if the CI value comes equal to 1 (CI = 1), the additive effect is indicated, which shows that no one of the two drugs interferes with the action of each other. To calculate the combination index, the cell migration assay was performed using varying concentrations of chitosan and insulin on HEKa cells, and further calculations were done using the given equation.

b $${\text{CI}} = \left( {\text{D}} \right){1}/\left( {{\text{Dx}}} \right){1} + \left( {\text{D}} \right){2}/\left( {{\text{Dx}}} \right){2}$$

c $${\text{Where}},{\text{ Dx}} = {\text{Dm }}\left[ {{\text{fa}}/{\text{fu}}} \right]^{{{1}/{\text{m}}}}$$

Here, (D)1 represents the chitosan concentration, whereas (D)2 denotes insulin concentration. The median effect equation (f) was used for the determination of single drug concentrations giving the same effect (Dx)1 and (Dx)2. The fa and fu give affected and unaffected cell fractions in the median dose, respectively, and are equal to 10^(y–intercept)/m^, where m indicates the slope median in the median effect plot of log (D) versus log (fa/fu).

### In vivo* experiment*

#### Experimental animal accusation and maintenance:

Five-week-old female Swiss albino mice were taken from the Animal Care facility of Chittaranjan National Cancer Institute. All animals were carefully maintained at an ambient temperature of 25 °C and a daily supply of healthy food and water according to the instruction of the Institutional Animal Ethics Committee. All experimental procedures were carried out in accordance with relevant guidelines and regulations approved by the Institutional Animal Ethics Committee of Chittaranjan National Cancer Institute (IAEC approved proposal no: IAEC-1774/BB-2/2018/10).

#### Third-degree Burn wound induction in mice

Five-week-old 40 female Swiss albino mice were chosen and acclimatized for one week in a new animal house. Before induction of the burn wound, hairs from the dorsal side of each animal were removed carefully. Mice were anesthetized by intraperitoneal (IP) injection of Ketamine (87 mg/kg b.w) and xylazine (13 mg/kg b.w) according to individual weight. Distilled water was boiled at 100 °C, and a 13 mm metal rod was placed on it for 1 min. A hot metal rod was placed on the shaved dorsal surface of each anesthetized mouse for 10 s.

#### Treatment and measurement of burn wound healing

All mice were caged individually after burn wound induction. After burn induction, the mice were randomly segregated into five groups, i.e., Control, ECNP, Insulin, ICNP, and Standard, each containing eight mice, and all experiments were repeated twice. All treatments were applied topically over the wound area for up to 20 days. Control groups were treated with saline, and the standard group received commercially available ointment Silverex. 100 µl of ECNP, insulin, and ICNP (each 60 µM) were applied topically over the wound. All groups received treatment up to the day of sacrifice.

The wound area was measured in regular intervals with a digital caliper by measuring the length (L) and width (W) of the wound. The wound area was analyzed by the formula Area (A) = $\pi /4 LW$. The formula analyzed the percentage of wound reduction. Wound reduction (%) = [(A_0 _− A_t_)/A_0_] × 100, where A_0_ is the initial wound area, and A_t_ is the final wound area.

#### Wound tissue collection and histological staining

Half of each group was randomly selected and sacrificed after 10 days of treatment, and the other half was sacrificed after 20 days. Wound tissue samples and plasma were collected from the freshly sacrificed animals. Mice were sacrificed by overdose of Ketamine (160 mg/kg) with xylazine (20 mg/kg) via intraperitoneal injection. Wound skin tissues were washed gently in PBS and submerged in 10% neutral-buffered formalin (NBF) overnight. Before block preparation, tissue sections were processed in 70% alcohol for 2 h, then in 90% alcohol for 1 h, acetone for 1 h, Xylene for 30 min, and finally in liquid paraffine overnight at 65 °C. Tissue blocks were prepared and sectioned into 5 µm thick slices. Tissue sections were placed over Poly-L-Lysine coated slides for histological staining. For Hematoxylin and Eosin staining, tissue sections containing slides were deparaffinized in Xylene for 15 min. Slides were rehydrated by sequential incubation in 100%, 90%, 70%, and 50% alcohol for staining with hematoxylin and eosin. Then, the slides were dehydrated in increasing alcohol concentration and mounted with a DPX (Dibutylphthalate Polystyrene Xylene) mounting medium. Finally, stained slides were observed under a microscope and photographed.

#### Masson's trichrome staining for collagen

In Masson's trichrome staining procedure performed after the sacrifice of the animals on the 20th day, skin tissue sections were initially deparaffinized using Xylene and sequentially rehydrated through incubations in 100%, 90%, 70%, and 50% alcohol for 5 min each. The sections were then stained with Weigert's iron hematoxylin for 10 min and subsequently subjected to Biebrich scarlet-acid fuchsin staining for 5 min, followed by treatment with phosphomolybdic–-phosphotungstic acid solution for 10 min and aniline blue staining for 10 min. Afterward, the slides were dehydrated, mounted, and observed under a microscope. This staining method enabled the visualization of collagen synthesis, with collagen fibers appearing blue, and allowed for the evaluation of the effects of chitosan insulin nano-formulations on collagen deposition in the skin tissues, contributing to the understanding of wound healing mechanisms.

#### Collection of plasma and analysis of cytokine

After 10 and 20 days of burn injury, blood was collected from each group of mice in EDTA (Ethylene diamine tetraacetate) coated vials. Collected blood was centrifuged at 1500 rpm for 10 min to separate the plasma and stored at − 80 °C until use. IL-6 and IL-10 ELISA (Enzyme-Linked Immunosorbent Assay) kits (BioLegend) were used to analyze the Pro and Anti-inflammatory cytokine levels. Before running the ELISA, the capture antibody was coated in a 96-well plate overnight at 4 °C. Blocking was performed, and standard solutions were prepared along with plasma samples, incubated for 1 h, and washed with wash buffer. Then, wells were sequentially incubated with detection antibody and Avidin-HRP (Avidin-Horseradish Peroxidase) solution. Wells were thoroughly washed after every incubation. TMB (Tetramethylbenzidine) substrate solution was added in the dark for 20 min, and the reaction was stopped by using a stop solution. Finally, absorbance was measured in a spectrophotometer at 450 nm within 15 min. The standard curve was prepared, and unknown concentrations were analyzed from OD (Optical Density) values.

#### Immunohistochemical staining of burn wound skin tissues

Skin tissue samples were collected from the burn wounds of experimental animals at 10 days post-injury. Tissue samples from different treated groups were obtained, including the control, chitosan nano-formulation (ECNP), insulin, standard drug, and chitosan insulin nano-formulation (ICNP) groups. The collected skin tissue samples were fixed in 10% formalin for 24 h and processed for paraffin embedding. Paraffin-embedded tissue blocks were sectioned into 5 μm thick slices using a microtome. The tissue sections were mounted on glass slides and subjected to deparaffinization and rehydration.

The tissue sections underwent antigen retrieval by heat-induced epitope retrieval using citrate buffer (pH-6.0) at 85 °C for 30 min. Endogenous peroxidase activity was blocked with hydrogen peroxide treatment. The Nrf-2 primary antibody (R&D Systems) was used in 1:200 dilution and incubated overnight at 4 °C. After washing; slides were incubated with HRP-tagged secondary antibody (Santa Cruz Biotechnology) for 30 min at room temperature. The sections were treated with a DAB (3,3′-diaminobenzidine) substrate, resulting in a brown color reaction at the site of Nrf-2 expression. This allowed for the visualization of Nrf-2-positive cells and tissues.

The stained tissue sections were observed under a light microscope. The staining intensity and distribution of Nrf-2 immunoreactivity were assessed and documented. Quantitative or qualitative analysis was performed by ImageJ, and statistical analyses were conducted to determine significant differences between the treated groups.

### Statistical analysis

Data are represented as mean ± SE. Statistical values were analyzed using analysis of variance (ANOVA) and multiple comparison tests using GraphPad Prism version 7.0 (GraphPad Software, La Jolla, CA, United States). A *p* value of < 0.05 was considered significant for all analyses.

## Results and discussions

### Structure and morphological studies

FE-SEM and Dynamic light scattering studies were used to study nanoparticle morphology and hydrodynamic size. The size of ECNP nanoparticles comes out to be ~ 25 nm (Fig. 1b), while that of ICNP nanoparticles is ~ 30 nm (Fig. 1c), and the particles come out to be spherical in FE-SEM. The hydrodynamic diameter of ECNP alone was slightly different from that of ICNP. At pH 6, the size of ECNP was 180.88 ± 20 nm, while ICNP was 187.47 ± 20 nm. At pH 7.4, ECNP was found to be 255.44 ± 20 nm, while for ICNP, the size was 289.20 ± 20 nm. The size comes out to be 133.27 ± 20 nm and 214.57 ± 20 nm for ECNP and ICNP, respectively, at a pH of 8. The size is 174.44 ± 20 nm for ECNP (Fig. 1d) and 119. 50 ± 40 nm for ICNP at pH 9 (Fig. 1e). A comparative DLS data at different pH is shown in (Supplementary file 1: Fig. S1a–h). There is also a change in Zeta potential with a change in pH. For ECNP, zeta potential comes out to be 10.9 ± 2.74, 25.9 ± 3.39, 26.7 ± 2.63, and 24.6 ± 3.1 at pH 6, 7.4, 8, and 9, respectively. Similarly, there is variation in zeta potential values of ICNP. It was found to be 28.8 ± 3.08, 23.2 ± 2.9, 23.4 ± 3.12, and 14.7 ± 2.5, respectively, indicating the formation of desired nanoparticles as mentioned in Supplementary file 1: Table S1.

### FTIR spectra showed conformational changes at the protein level due to chitosan-insulin interactions

**Fig. 2:**
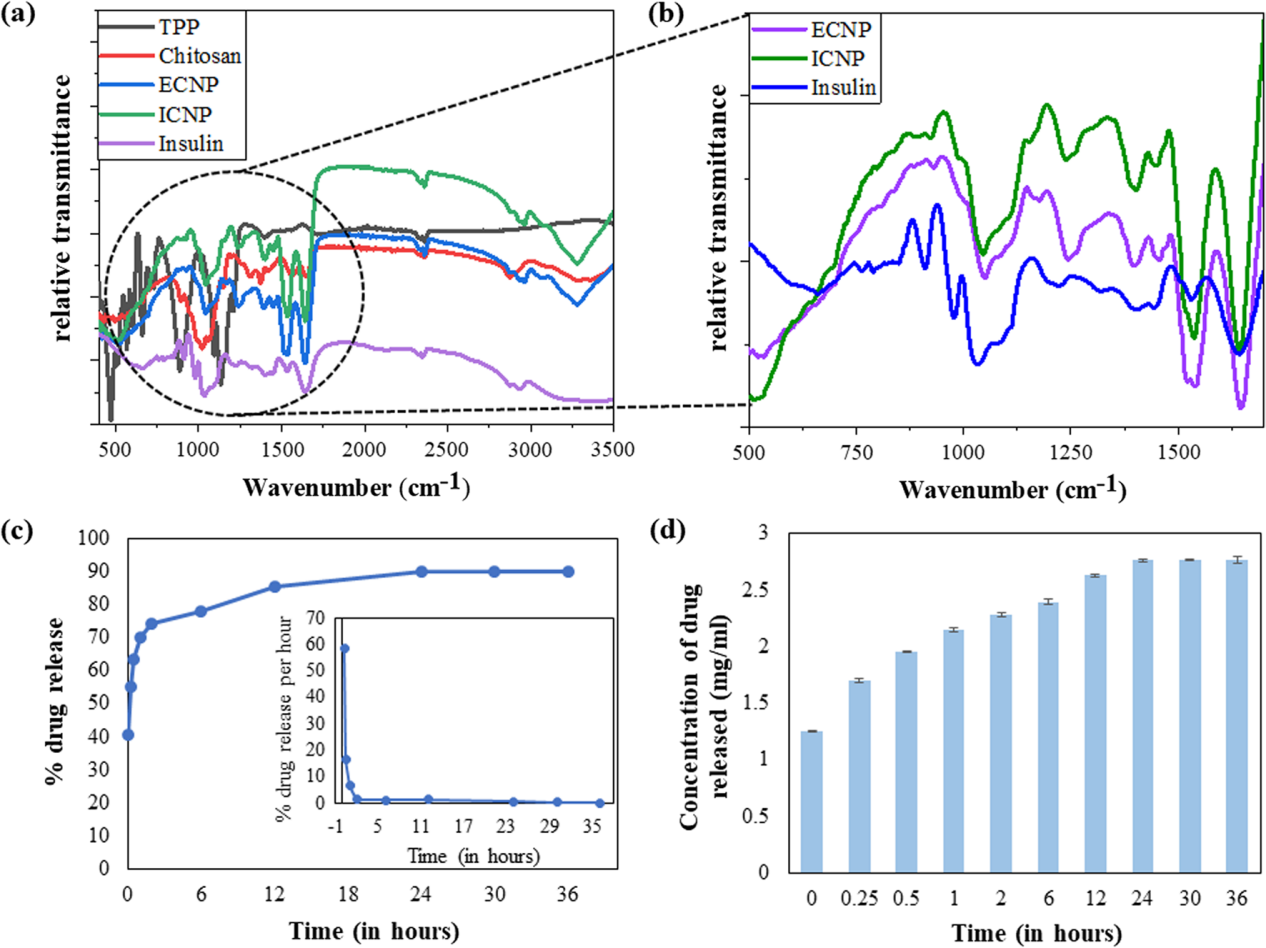
The figure shows the spectroscopic studies to monitor structural changes in insulin protein after interaction with chitosan and the drug release studies from ICNPs. The plots show the FTIR spectroscopic studies of TPP, chitosan, ECNP, insulin, and ICNP, indicating the changes in the wavenumber of characteristic bonds, confirming the interactions between ECNP and insulin at different scale bar **a** 500–4000 cm^−1^ and **b** 500–1500 cm^−1^ (fingerprint region). The release kinetic studies from ICNP nanoparticles **c** Plot for the % of total drug release during the time span of 36 h from ICNP (Inlet shows the plot for the release kinetics indicating the % drug release per h). **d** The graph provides information on the drug release in mg/ml from ICNP

FTIR was used to determine the interactions between TPP, chitosan, and insulin, and studies were done for TPP, chitosan, insulin, ECNP, and ICNP. Firstly, the peak indicating N–H stretch appeared at 3294 cm^−1^ and 3282 cm^−1^ in chitosan and ECNP, respectively, whereas for ICNP, it was found at 3272 cm^−1^ and 3282 cm^−1^ [60]. Then, a peak at 3054 cm^−1^ was observed in ECNP and ICNP, indicating aromatic C-H stretching [61]. Similarly, for aliphatic C-H stretching, the peak was at 2878 cm^−1^ in chitosan, 2867 cm^−1^, 2950 cm^−1^ in ECNP, 2826 cm^−1^ in insulin, and 2878 cm^−1^ and 2950 cm^−1^ in ICNP [61]. The peak indicating C=O stretch (Amide-I, O=C–NHR) was found at 1642 cm^−1^ in chitosan, ECNP, ICNP, and 1641.99 cm^−1^ in insulin [62], while for N–H bend (Amide-II) it appears at 1561 cm^−1^ for chitosan, 1533.65 cm^−1^ for insulin and at 1536 cm^−1^ for both ECNP and ICNP [63]. In addition to these peaks, a peak at 1431 cm^−1^ in insulin and 1447 cm^−1^ in ECNP and ICNP was observed exhibiting the S=O bond [44]. O–H bending was observed at 1323 cm^−1^ and 1375 cm^−1^ in chitosan, 1399 cm^−1^ in ECNP, 1336 cm^−1^ in insulin and 1393 cm^−1^ in ICNP [64]. The peak at 1241 cm^−1^ in ECNP and ICNP while at 1215 cm^−1^ and 1294 cm^−1^ shows the bond for C–O stretching [65]. Similarly, C-N stretching was observed only in insulin at 1104 cm^−1^ [66]. The symmetric and asymmetric vibrations for O–P=O were found only in TPP at 1167 cm^−1^, and the PO_3_ symmetric and asymmetric stretching vibrations were observed at 1046 cm^−1^ in chitosan, ECNP, and ICNP [67]. For C-O bending, the peak appeared at 930 cm^−1^ in ECNP, 930 cm-1 and 973 cm-1 in insulin, and 25 cm^−1^ and 988 cm^−1^ in ICNP [65]. The stretching vibration of the P-O-P bridge was observed alone in TPP at 887 cm^−1^ [67]. NH_2_ bending vibration was at 803 cm^−1^ and 872 cm^−1^, while C–O–O bending was at 746 cm^−1^ only in insulin. Similarly, both the C=S stretch and S–S stretch were observed at 651 cm^−1^, 682 cm^−1^, and 551 cm^−1^, respectively [8] as shown in (Fig. 2a and b). A comparative FTIR data of TPP, chitosan, insulin, ECNP, and ICNP is given in Table 1.

**Table 1:** The comparative wavenumber values indicated by FTIR (in the range 400–4000 cm^−1^) of TPP, chitosan, ECNP, insulin, ICNP showing the position of different functional groups present, which coins the interaction amongst these solutions indicating the formation of insulin loaded chitosan nanoparticles

Functional groups	TPP	Chitosan	ECNP	Insulin	ICNP	References
N–H stretch (Amide-I, O=C–NHR)	–	3294	3282	–	3282 3272	[60]
Aromatic C–H stretching	–	–	3054	–	3054	[61]
Aliphatic C–H stretching	–	2878	2950 2867	2826	2950 2878	[61]
C=O stretch (Amide-I, O=C–NHR)	–	1642	1642	1641	1642	[62]
N–H bend (Amide-II NH_2_)	–	1561	1536	1533.65	1536	[63]
S=O bond	–	–	1447	1431	1447	[44]
O–H bending	–	1375 1323	1399	1336	1393	[64]
C–O stretching	–	–	1241	1294 1215	1241	[65]
O–P=O Symmetric and asymmetric stretching vibrations	1167	–	–	–	–	[67]
C–N stretching	–	–	–	1104	–	[66]
PO_3_ Symmetric and asymmetric stretching vibrations	–	1046	1046	–	1046	[67]
C–O bending	–	–	930	973 930	988 925	[65]
Stretching vibrations of P–O–P bridge	887	–	–	–	–	[67]
NH_2_ bending vibrations	–	–	–	872 803	–	[8]
C–O–O bending				746		[8]
C=S (stretch)	–	–	–	651 682	–	[8]
S–S stretch	–	–	–	551	–	[8]

### Protein loading and release kinetics studies

The insulin protein loading and release kinetics of ICNP were studied, where the amount of insulin loaded was 88.725 ± 0.295% in the ECNP particles. The release kinetics of insulin were studied from ICNP particles at the physiological pH of 7.4 and the physiological temperature of 37 °C. The particles were loaded into the dialysis membrane, and the release kinetics was observed by taking the OD values at 595 nm after particular time intervals for a period of 36 h. It was observed that burst drug release was observed in the initial 2 h, followed by a sustained drug release till 12 h. Most of the insulin was released within 36 h. The percentage of insulin released during the first 2 h is 73.98 ± 0.019%, while by the end of 36 h, the total insulin released was 89.84 ± 0.01% of the loaded one, and the data is shown in (Fig. 2c, d). This indicates the high drug delivery potential of ICNP nano-formulations within less time intervals, making them efficient and effective drug delivery agents.

### Cell viability studies

**Fig. 3:**
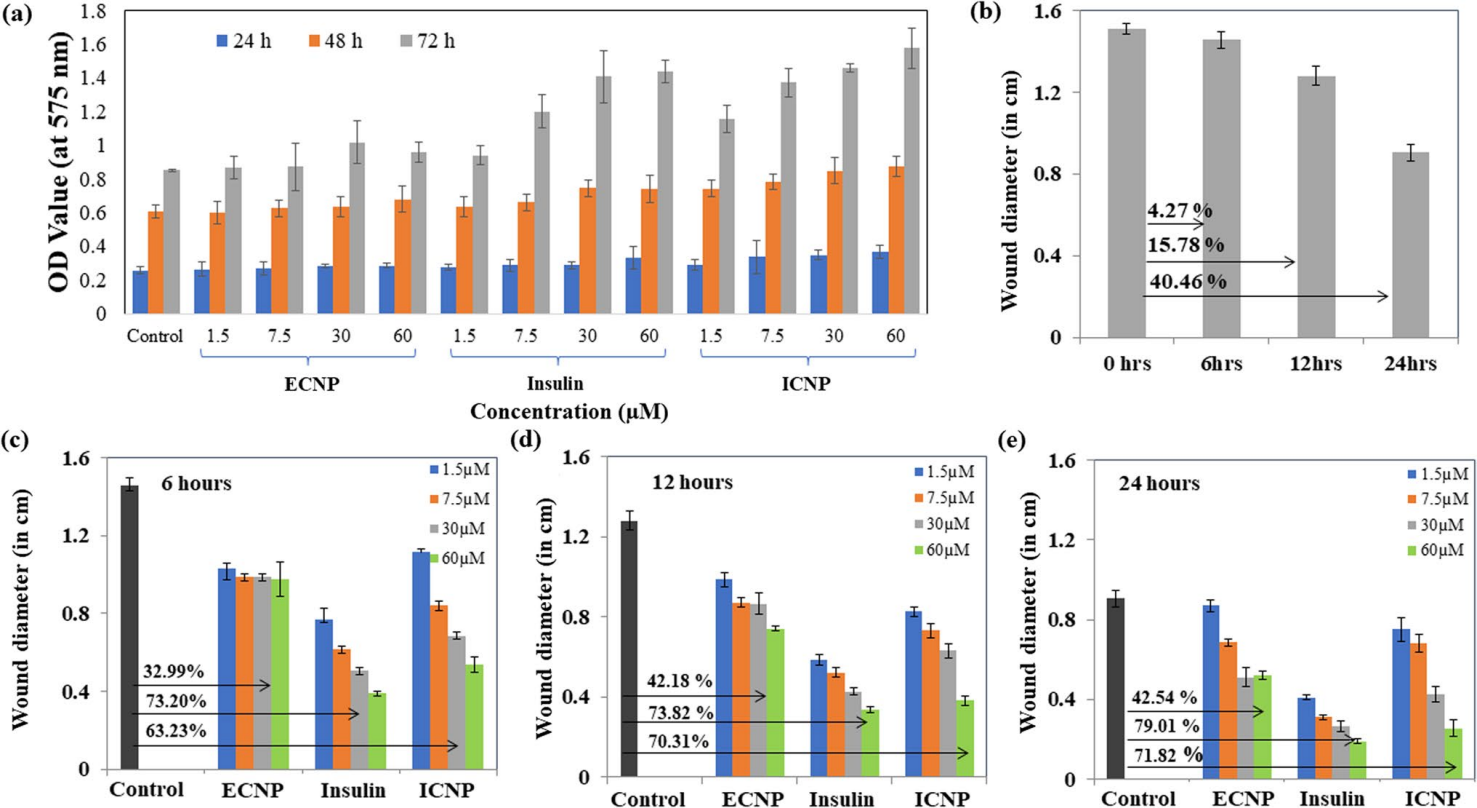
This figure indicates the cell viability studies and migration studies (n vitro) for confirming the wound healing abilities of ICNP **a** The graph shows the treatment of HEKa cells for 24 h, 48 h, and 72 h with 1.5 µM, 7.5 µM, 30 µM, and 60 µM of ECNP, insulin, and ICNP to check their biocompatibility, and the data indicates that they are not toxic to the normal skin cells. Promotion and monitoring of in vitro wound recovery using ECNP, insulin, and ICNP at different concentrations and time intervals, indicating the ICNP nanoparticles are efficient wound healing agents, and the healing effect increases with concentration and time. The figures show a comparative study of all three formulations of varying concentrations of 1.5 µM, 7.5 µM, 30 µM, and 60 µM after **b** 6 h of treatment, **c** 12 h of treatment, and **d** 24 h of treatment to determine the effect in wound healing with time and concentration

After incubating the HEKa cells with varying concentrations (1.5, 7.5, 30, 60 µM) of ECNP, Insulin, and ICNP for 24 h, 48 h, and 72 h, the cell viability was determined using an MTT assay. As is known, the cell viability using MTT assay is highly dependent upon the mitochondrial activity of the cells. The results show that none of the three components, including ECNP, insulin, and ICNP, are toxic to the HEKa cell line; instead, they can potentially enhance the cell division of normal human epidermal keratinocyte cells and thus can be used for wound healing applications. As we go on increasing the concentration and time duration of incubation for all samples, there is a tremendous improvement in the growth rate of cells, and it was maximum for the highest concentration, that is, 60 µM and maximum after 72 h incubation period. The % increase in cell growth is 143.807 ± 3.75, 146.71 ± 5.86, and 185.14 ± 12.17 after 24, 48, and 72 h as compared to the control, which is assumed to be 100% for each time point with varying absorbance values, and the studies are done in relevance to it. The variation in OD value for different concentration of each compound was plotted against the time and is shown in Fig. 3a. A comparative table for varying values of OD are given in Supplementary file 1: Table S2.

### in vitro HEKa cell migration studies

ICNP-treated cells show enhanced HEKa cell migration compared to untreated cells (control), ECNP, and insulin-treated cells. There is an enhancement in the extent of cell division and growth with the increase in time and concentration. After treatment with ICNP for 6, 12, and 24 h, the cells show a gradual decrease in the scratch diameter with increased time duration. The percentage of the gap left after 6, 12, and 24 h of treatment with ICNP is 76.63 ± 2.30%, 64.45 ± 2.88%, and 96.13 ± 2.30%, respectively, at 1.5 µM; 57.732 ± 2.30%, 57.03 ± 3.46% and 75.13 ± 4.61% respectively at 7.5 µM; 47.07 ± 1.73%, 49.21 ± 3.46% and 46.96 ± 2.88% respectively at 30 µM; 36.76 ± 4.04%, 29.68 ± 2.30% and 28.17 ± 4.04% respectively at 60 µM. In the case of insulin, the percentage of the gap left between the wound scratch is more in comparison to that of ICNP. The HEKa cells after treatment with insulin show the percentage change in scratch diameter after 6, 12, and 24 h as 52.92 ± 5.77%, 45.70 ± 2.88%, and 45.30 ± 1.15%, respectively, at a concentration of 1.5 µM; 42.26 ± 1.73%, 40.62 ± 2.30%, and 34.25 ± 1.15% respectively at 7.5 µM. The variation in wound scratch diameter is found to be 34.70 ± 1.73%, 33.20 ± 1.73%, and 29.28 ± 1.73%, respectively, for 30 µM concentration; and 26.80 ± 1.15%, 26.17 ± 1.73% and 20.99 ± 1.15% respectively for 60 µM of insulin. In the case of ECNP, migration of cells is observed compared to the untreated cells. ECNP-treated cells at a concentration of 1.5 µM show the % change in wound diameter after 6, 12, and 24 h as 70.99 ± 5.77%, 76.95 ± 2.30%, and 96.13 ± 5.77 respectively. At 7.5 µM concentration, the change with increasing time interval is 67.69 ± 1.73%, 67.57 ± 1.73%, and 75.69 ± 1.73%, respectively. The values come out to be 67.69 ± 1.73%, 67.57 ± 1.73%, and 56.35 ± 1.15, respectively, after 6, 12, and 24 h, while for 60 µM concentration % change is 67.01 ± 8.66%, 57.81 ± 1.15% and 57.40 ± 2.30% respectively. The comparative data of wound migration for control and under the influence of varying concentrations of ECNP, insulin, and ICNP is shown in (Fig. 3b–e) and (Supplementary file 1: Figs. S2–S4). For evaluating the statistical significance of the data, the p values were calculated for % variation in wound diameter after treatment with varying concentrations of ECNP, insulin, and ICNP (1.5, 7.5, 30 and 60 µM) after 6 h, 12 h, and 24 h respectively and the statistical significance of data is considered when *p *< 0.05 and is given in Table S3.

### Combination index of chitosan and insulin

For the calculation of the combination index, Dm was calculated by using m and y from (Supplementary file 1: Fig. S5a) for chitosan and (Supplementary file 1: Fig. S5b) for insulin after 6 h, (Supplementary file 1: Fig. S5c) for chitosan and (Supplementary file 1: Fig. S5d) for insulin after 12 h, and (Supplementary file 1: Fig. S5e) for chitosan and (Supplementary file 1: Fig. S5f) for insulin after 24 h. The calculated combination index values for the chitosan and insulin exhibit a synergistic effect. After calculating the combination index by varying the concentrations of both chitosan and insulin, the values come out to be less than one, representing the synergism between the two drugs; that is, they enhance each other's activity by working cordially. These results are in resemblance with the ones obtained earlier from cell viability and cell migration assays performed in vitro*,* indicating the potential therapeutic role of chitosan and insulin in enhanced wound healing applications. The data is shown in Supplementary file 1: Table S4.

### Chitosan insulin nano-formulation accelerates burn wound healing through collagen deposition and tissue remodeling

**Fig. 4:**
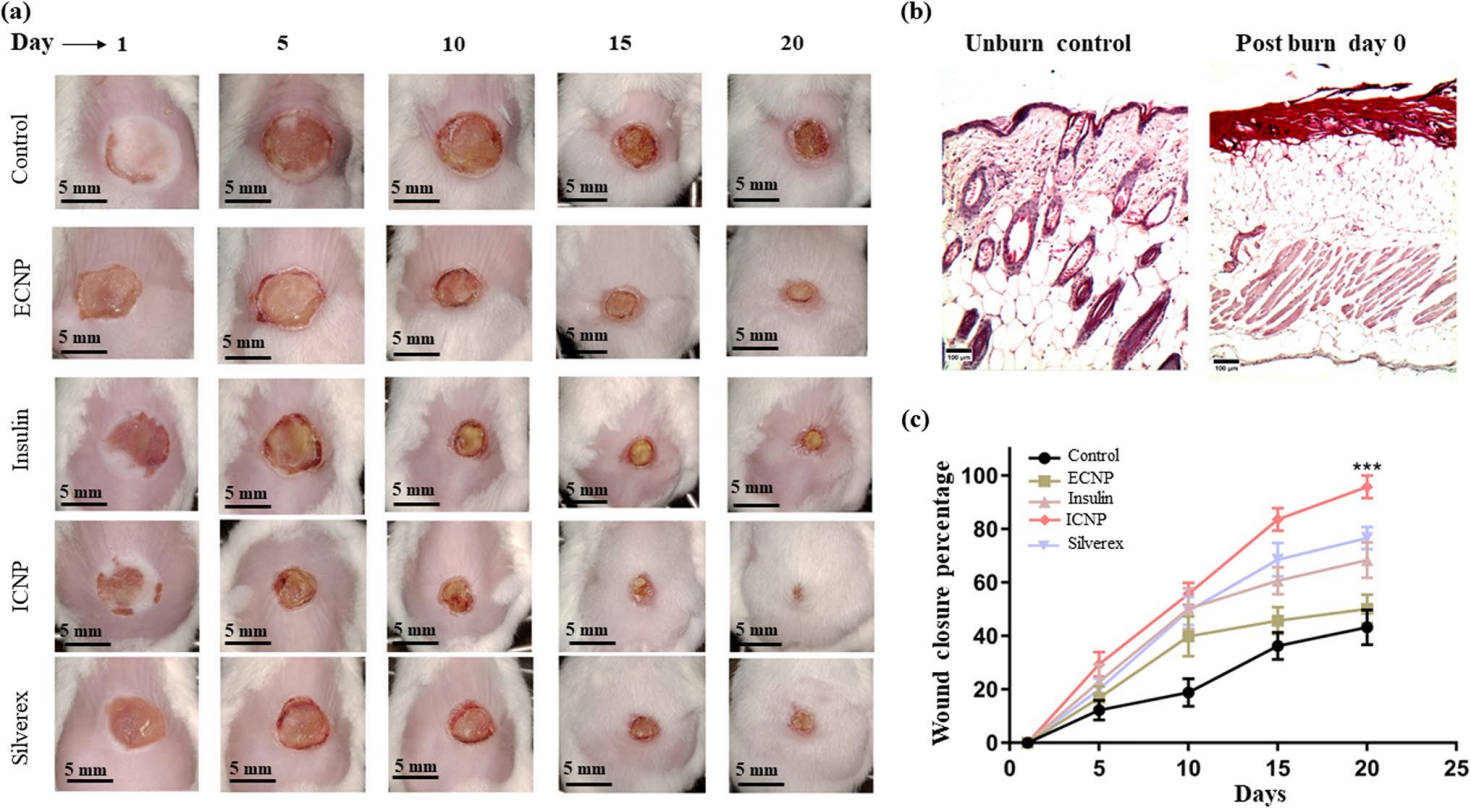
Representative images of day-wise burn wound closure images of control and all treated groups **a** Hematoxylin and Eosin staining of post-burn day 0 and unburn control mice skin. Day 0 burn wound skin showing the development of third-degree burn wound indicated by completely damaged epidermis, dermis, and hypodermis **b** Percentage of burn wound closure up to 20 days **c** Data represents as mean ± SEM; ***p < 0.001; *represents a significant difference from the control group)

**Fig. 5:**
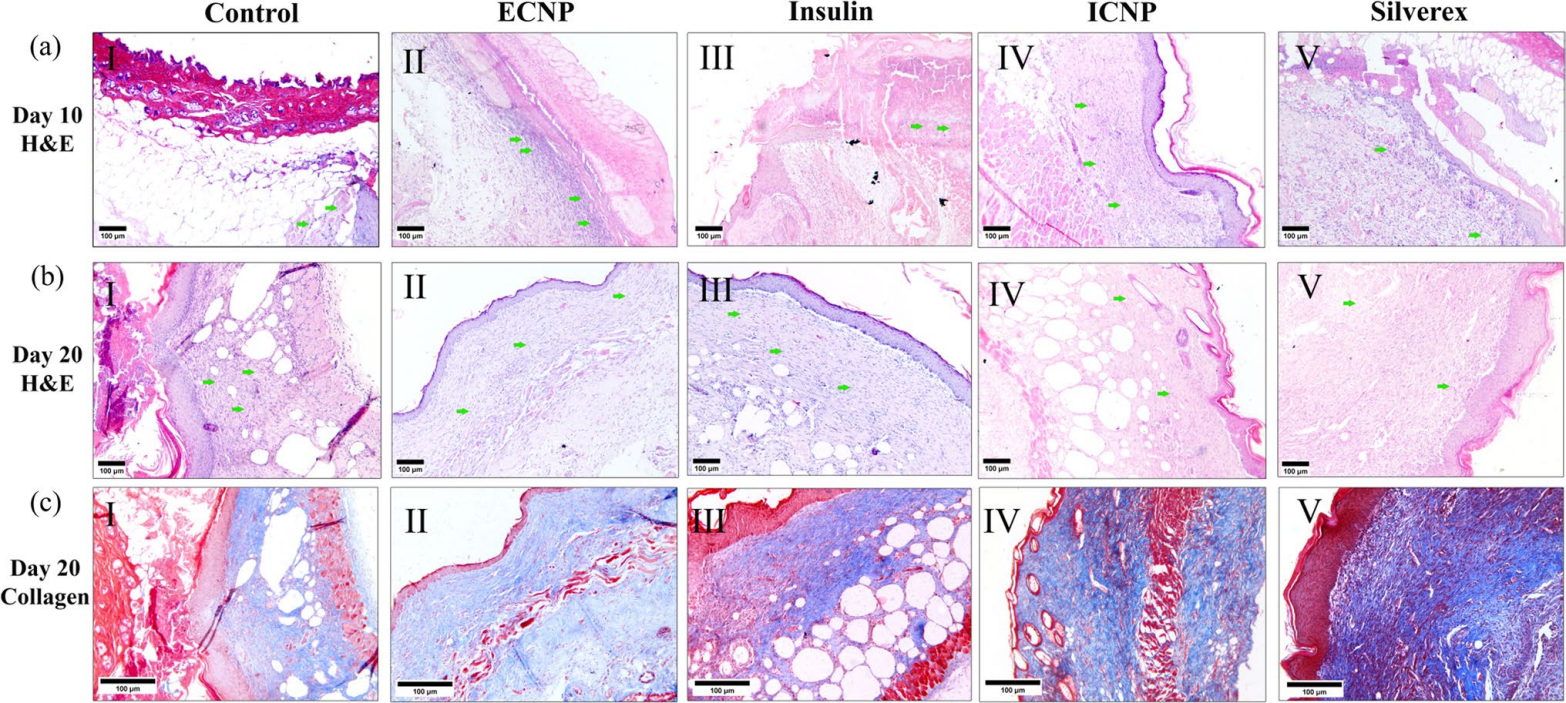
The figure depicts tissue histology and collagen deposition during burn wound healing Hematoxylin and eosin-stained post-burn **a** day 10, **b** day 20, and **c** collagen-stained day 20 wound skin histology images. Green arrows indicate inflammatory cell infiltration. All H&E images were taken at 100X magnification, and collagen staining images at 200X magnification (Scale bar 100 µm)

Burn wound closure was monitored over 20 days starting from wound induction (Day 0) (Fig. 4a) shows representative images of the wounds during the treatment period in different groups, following confirmation of third-degree burn wound histology with healthy mice skin histology on day 0 (Fig. 4b). No significant wound closure was observed among the treatment groups for up to 5 days, but only ICNP treated group significantly reduced the wound by 29.3 ± 4.63% (Fig. 4c). Significant wound closure was observed in all treatment groups compared to the control group from 10 days of treatment. The degree of wound closure was less in the control and ECNP groups, whereas insulin, standard, and ICNP-treated groups reduced the wound area by 50.26 ± 7.65%, 49.39 ± 5.29%, and 55.66 ± 4.17%. A similar pattern was observed after 20 days of treatment. The ICNP-treated group displays more significant wound contraction of 95.77 ± 4.26% than the insulin and standard group, i.e., 68.38 ± 6.62% and 76.57 ± 4.14%. Control and ECNP groups showed less wound reduction, about 43.25 ± 6.56% and 50.13 ± 5.39%. 10-day post burn own skin tissue histology images (Fig. 5a) showed control and ECNP group dominated with inflammatory cell-rich tissue. Wound eschar started to separate from the wound bed in Insulin and standard groups, and epidermal keratinocyte migration started beneath the eschar to re-epithelization of the wound area. The ICNP-treated group was observed to have less inflammatory cell infiltration, a complete absence of wound eschar, and moderate reepithelization. On day 20, the control group still exhibited wound eschar, enhanced inflammatory cells, and re-epithelization in a small area (Fig. 5b); however, ECNP, insulin, and the standard group were characterized by increasing epidermal thickness. Moreover, Well-repaired hair follicles were observed in the ICNP-treated group, indicating accelerated wound healing and re-epithelization.

In Masson's trichrome collagen staining, collagens are stained blue, whereas muscle and cytoplasm are stained red. The staining assessed the collagen deposition during the granulation tissue formation. The intensity of blue staining corresponds to the relative quantity of new collagen fiber deposition. On 20-day skin tissue collagen staining, less blue stained area and intensity were observed in control and ECNP groups (Fig. 5c). The rate of collagen formation was comparatively higher in insulin and standard-treated groups, but the presence of reddish areas in collagen indicates burn-induced denatured collagen fibers. The highest new collagen fiber deposition was observed in the ICNP-treated group, marked by a high-intensity blue-stained area. This suggests that new collagen was rearranged during wound healing, and the ICNP-treated group showed this in the shortest time.

### Chitosan insulin nano-formulation regulates pro and anti-inflammatory cytokines

**Fig. 6:**
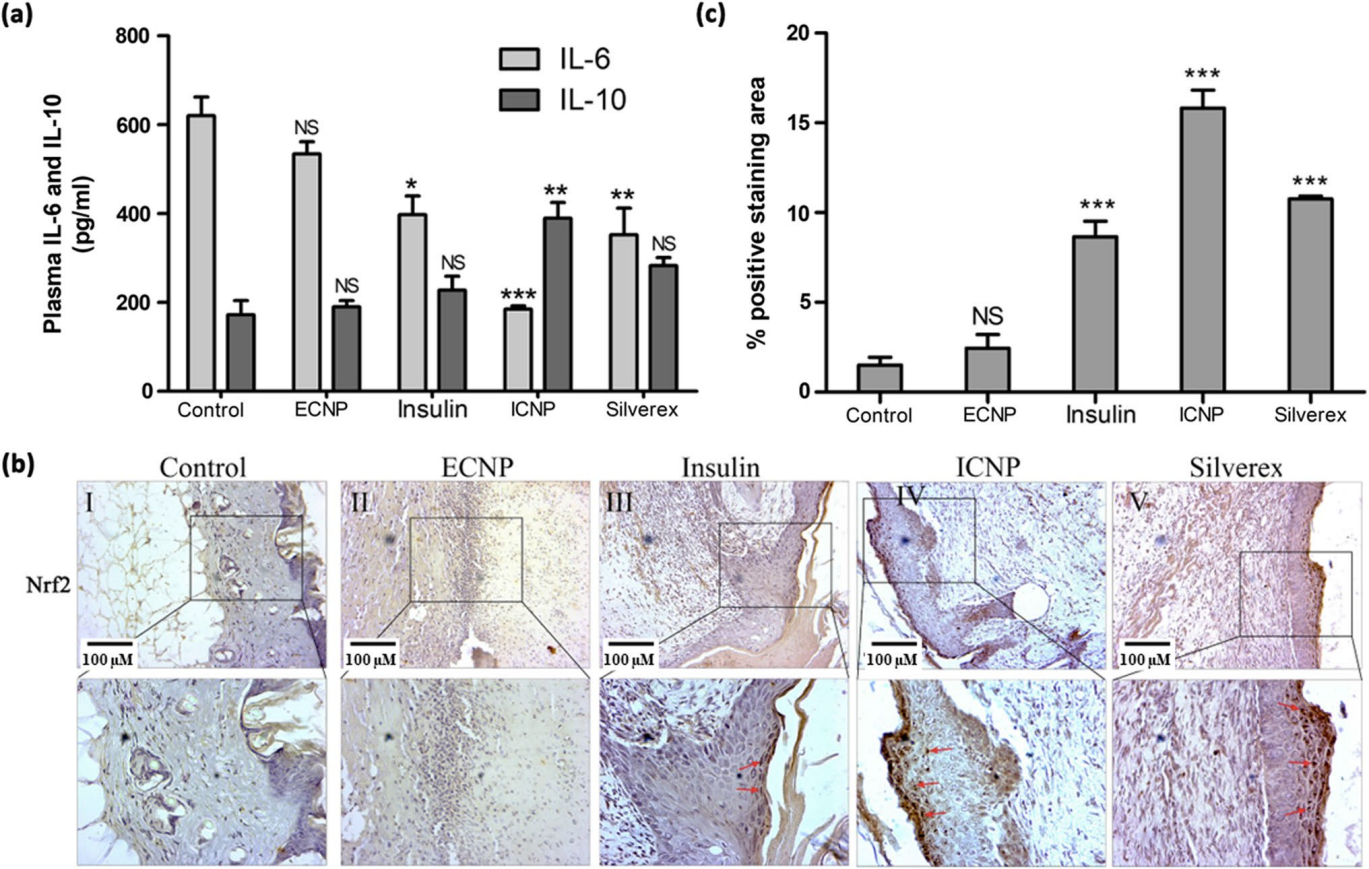
The figure provides information about the regulation of inflammatory cytokines during burn wound healing along with Immunohistochemical (IHC) staining of Nrf-2 expression in 10-day post-burn wound tissue sections **a** Post-burn injury plasma IL-6 and IL-10 levels were collected on day 10 to analyze the inflammatory phases. **b** Immunohistochemical (IHC) staining of Nrf-2 expression in 10-day post-burn wound tissue sections. Representative images show Nrf-2 immunoreactivity in different treatment groups, including control, chitosan insulin nano-formulation (ECNP), insulin, standard drug, and chitosan insulin nano-formulation (ICNP). The ICNP-treated group displays significantly higher Nrf-2 expression compared to other groups. Red arrows indicate positive Nrf-2 staining, **c** Quantitative analysis of positive stained areas was performed using ImageJ software for Nrf-2 immunohistochemical staining in burn wound tissues. Data represented as mean ± SEM; *p < 0.05, **p < 0.01, ***p < 0.001; *represents a significant difference from the control group; NS (Nonsignificant); Scale bar 100 µm

Pro-inflammatory and anti-inflammatory cytokine levels in plasma were evaluated 10 days after burn injury. The control and chitosan nano-formulation (ECNP)-treated groups exhibited high levels of plasma IL-6 (620.60 ± 41.58 pg/ml and 534.35 ± 27.37 pg/ml) and relatively low levels of plasma IL-10 (172.50 ± 31.82 pg/ml and 190.00 ± 14.14 pg/ml). Treatment with insulin and a standard drug significantly reduced IL-6 levels (397.50 ± 42.43 pg/ml and 352.50 ± 60.10 pg/ml) while increasing plasma IL-10 levels (227.50 ± 31.82 pg/ml and 282.75 ± 18.03 pg/ml). Furthermore, topical administration of the chitosan insulin nano-formulation (ICNP) markedly decreased IL-6 levels to 185.00 ± 7.07 pg/ml, accompanied by an elevated IL-10 plasma content of 389.95 ± 35.28 pg/ml. The elevated IL-6 levels observed in the control and ECNP-treated groups indicate an ongoing pro-inflammatory response. However, treatment with insulin or the standard drug showed a significant reduction in IL-6 levels and an increase in IL-10, indicating the ability of these interventions to suppress excessive inflammation. The changes in mean plasma IL-10 levels for ECNP, Insulin, and Silverex groups were not significant, but the ICNP group showed significant change compared to the control group. ICNP showed better efficacy than insulin or the standard drug by significantly reducing IL-6 levels and elevating IL-10 levels, showing the best modulation of pro-inflammatory and anti-inflammatory cytokine profiles (Fig. 6a).

The comparison of plasma IL-6 and IL-10 levels among the treatment groups at day 20 reveals the efficacy of various interventions (Supplementary file 1: Fig. S6). Compared to the control group, which had IL-6 levels of 267 ± 35.7 pg/ml, the ECNP group exhibited a slightly lower concentration at 221 ± 29.9 pg/ml. However, it's noteworthy that insulin and the standard drug group significantly reduced IL-6 levels, recording values of 139.7 ± 22.4 pg/ml and 112.3 ± 27.3 pg/ml, respectively. Remarkably, the ICNP-treated group demonstrated the most substantial decrease in IL-6 levels, reaching as low as 107.4 ± 25.4 pg/ml. Also, the plasma IL-10, the control group, had levels of 152.3 ± 22.6 pg/ml, and the ECNP group showed a similar concentration at 145.7 ± 19.3 pg/ml. Once again, both insulin and the standard drug group displayed decreased IL-10 levels, measuring 101.1 ± 21.6 pg/ml and 95.4 ± 30.5 pg/ml, respectively. In stark contrast, the ICNP-treated group exhibited the most remarkable drop in IL-10 levels, registering as low as 89.8 ± 20.8 pg/ml.

The observations at day 20 point towards a notable trend in the cytokine levels across all treatment groups. The IL-6 levels in the insulin, Silverex, and ECNP groups have decreased to what can be considered normal levels, signifying the resolution of the earlier pro-inflammatory response. Correspondingly, the levels of IL-10, which had been balancing the pro-inflammatory effects of IL-6, have also decreased notably in these groups. Interestingly, the control and ECNP groups still exhibit relatively higher IL-6 levels at day 20. However, these elevated IL-6 levels are somewhat compensated for by a slight increase in IL-10 levels. This might suggest a normal systemic response that occurs independently of any treatment and could be attributed to the ongoing wound-healing process.

### Chitosan insulin nano-formulation regulates Nrf-2 expression

Immunohistochemical (IHC) staining analysis revealed distinct Nrf-2 expression patterns among the treated groups (Fig. 6b). Quantification of positive Nrf-2 staining areas in burn wound tissues was performed using ImageJ software. Multiple images were captured at a high-power field from each treatment group, and the positive staining areas were calculated. The data were plotted in a bar diagram to illustrate the comparative Nrf-2 expression levels among the groups (Fig. 6c). In the control and ECNP groups, Nrf-2 expression levels in the IHC staining were very low compared to the other groups. This suggests that the control and ECNP treatments had a limited impact on activating the Nrf-2 pathway. In contrast, the insulin and standard drug groups exhibited moderate Nrf-2 expression in the IHC staining analysis. The ICNP-treated group displayed a significantly higher Nrf-2 expression in the IHC staining than all other groups. This suggests that the chitosan insulin nano-formulation, ICNP, effectively upregulates Nrf-2 expression, potentially enhancing the cellular defense mechanisms against oxidative stress and countering inflammation.

Insulin is known to have anti-inflammatory properties and can modulate the inflammatory response. Similarly, Nrf-2 plays a crucial role in suppressing inflammation by controlling the expression of antioxidant and detoxifying enzymes. The increased expression of Nrf-2 in the ICNP-treated group, as demonstrated by IHC staining, suggests that ICNP may regulate inflammation by activating Nrf-2. The table for checking the statistical significance of IL-6, IL-10and Nrf-2 data is given as Table S5.

## Conclusion

Burn wound healing poses a significant challenge in healthcare due to slow re-epithelialization and high susceptibility to microbial infection, ultimately affecting over 10 million people annually, as per the WHO report. Addressing this issue is crucial to alleviate the burden on the health sector, and protein-based nano-formulations are gaining attention depending on their characteristic features and potential to cure injuries. Insulin promotes healing in normal and diabetic conditions, but the potential and mechanism behind burn wound healing are yet to be explored. Insulin-based nano-formulations outperform conventional insulin due to high stability, enhanced solubility, improved bioavailability, high entrapment efficiency, and controlled release. This study highlights a potential nano-formulation involving chitosan-insulin nanoparticles as advanced therapeutic agents for burn wound healing and tissue regeneration.

Different techniques were used to monitor insulin and chitosan interactions, followed by their biocompatibility and wound healing studies in vitro and in vivo models. The enhanced cell migration observed in ICNP-treated cells suggests their role in promoting cell proliferation crucial for wound healing. The combination index calculations affirmed the synergistic effect of chitosan and insulin. In the animal burn wound model, the ICNP-treated group exhibited a remarkable 29.3% reduction in the wound area compared to other groups in the initial days of treatment, signifying its early efficacy. Subsequently, all treatment groups displayed differential wound closure compared to the control group after 10 days of treatment, while the ICNP-treated group demonstrated the highest rate of wound contraction of 95.77% after 20 days. Histological examinations after 10 and 20 days showed the least inflammatory cell infiltration, the complete absence of wound eschar, and significant reepithelization in the treated group. Masson's trichrome collagen staining indicated enhanced collagen fiber deposition in the ICNP-treated group, as shown by the intensive blue-stained area, indicating its potential in collagen synthesis during wound healing.

Further, the cytokine analysis on day 10 demonstrated that ICNP effectively suppressed pro-inflammatory cytokine IL-6 while elevating anti-inflammatory cytokine IL-10, and this trend persisted till day 20, showing its superior modulation of pro-inflammatory and anti-inflammatory cytokine profiles, while control and ECNP groups at day 20 indicated an ongoing pro-inflammatory response. Immunohistochemical staining revealed that ICNP significantly upregulated Nrf-2 expression compared to all other groups on day 10 of treatment, suggesting enhanced cellular defense against oxidative stress and inflammation. Nrf-2 is pivotal in regulating intracellular redox homeostasis by increasing cytoprotective gene expression, and its activation accelerates diabetic wound healing by facilitating diabetes-mediated oxidative stress and inflammation [27]. Considering that insulin possesses anti-inflammatory properties, the increased Nrf-2 expression in the ICNP-treated group indicated that ICNP regulates inflammation through Nrf-2 activation. Few studies show the protective roles of Nrf-2 in burn trauma-induced intestinal injury and burn-induced cardiac dysfunction [68, 69] as well as the role of topical insulin [33] or insulin nanoparticles [36] in burn wound healing, but none has implicated the role of insulin in activation of Nrf-2 pathway. Further, different formulations are being developed for burn wound healing, including the topical application of simvastatin on the burn wound Wistar rat model, and it was found to promote healing by modulating Akt/mTOR signaling pathway along with increased CD31 VEGF levels. Similarly, when the simvastatin was incorporated with bone marrow-derived mesenchymal stem cells, the outcomes were better and showed synergistic effect in enhanced re-epithelialization, higher wound closure area, enhanced collagen deposition, and epidermal regeneration and followed the same Akt/mTOR pathway for healing burn injuries [7, 33].

Our study showed effective modulation of inflammatory phases by activating the Nrf-2 pathway, underscoring its promise as a therapeutic intervention in burn care and management. This field holds remarkable potential to improve the lives of burn injury sufferers. The excellent drug loading and release efficiency and their potential in treating burn wounds in vitro and in vivo hold great value and can serve as a catalyst in burn wound healing research. Further, the global market of protein-based nanoformulations is rapidly expanding and is projected to double from $14 billion in 2020 to $28 billion by 2025, indicating its vast potential in the near future [70]. We firmly believe that the wound healing and anti-inflammatory properties of these particles and the key findings of this report will have a lasting impact, fostering innovation and ability to inspire future research in the field of burn wounds across the research community, ultimately improving and saving the lives of patients fighting against burn injuries.

### Supplementary Information

Supplementary file1 (DOCX 3082 KB)

## Data Availability

The authors declare that the data supporting the findings of this study are available within the paper and its Additional files. Should any data files be needed in any other format, they are available from the corresponding author upon reasonable request.
