# Supplementary material for: Chitosan-insulin nano-formulations as critical modulators of inflammatory cytokines and Nrf-2 pathway to accelerate burn wound healing
Source: Discov Nano. 2023 Dec 12;18(1):154. doi: 10.1186/s11671-023-03941-2 (PMC10716098; doi:10.1186/s11671-023-03941-2)
Supplement: Supplementary file 1 — Supplementary file1 (DOCX 3082 KB) [file 11671_2023_3941_MOESM1_ESM.docx]

**Chitosan-insulin nano-formulations as critical modulators of inflammatory cytokines and Nrf-2 pathway to accelerate burn wound healing**

Deepinder Sharda^1#^, Sandip Ghosh^2#^, Pawandeep Kaur^1^, Biswarup Basu^2^*, Diptiman Choudhury^1,3^*

# Authors contributed equally

1; Department of Chemistry and Biochemistry, Thapar Institute of Engineering and Technology, Patiala, 147004, Punjab, India

2; Department of Neuroendocrinology & Experimental Hematology, Chittaranjan National Cancer Institute, Kolkata, 700026

3; Centre of Excellence for Emerging Materials, Thapar Institute of Engineering and Technology, Patiala, Punjab- 147004, India.

***Corresponding E-mail**: [biswarup.basu@gmail.com](mailto:biswarup.basu@gmail.com), [diptiman@thapar.edu](mailto:diptiman@thapar.edu) ;

*Corresponding Phone: +91-8800883257, +91-8196949843


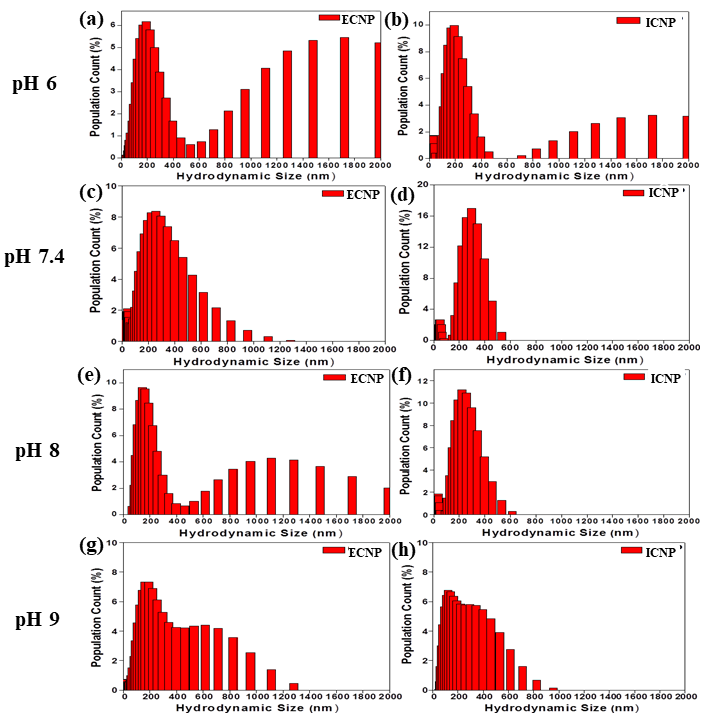


**Figure S1.** The figure represents the variation in size of nanoparticles (both empty chitosan nanoparticles (ECNP) and insulin-loaded chitosan nanoparticles (ICNP)) with slight variation in pH (a) ECNP and (b) ICNP at pH 6, (c) ECNP and (d) ICNP at pH 7.4, (e) ECNP and (f) ICNP at pH 8 and (g) ECNP and (h) ICNP at pH 9 respectively.


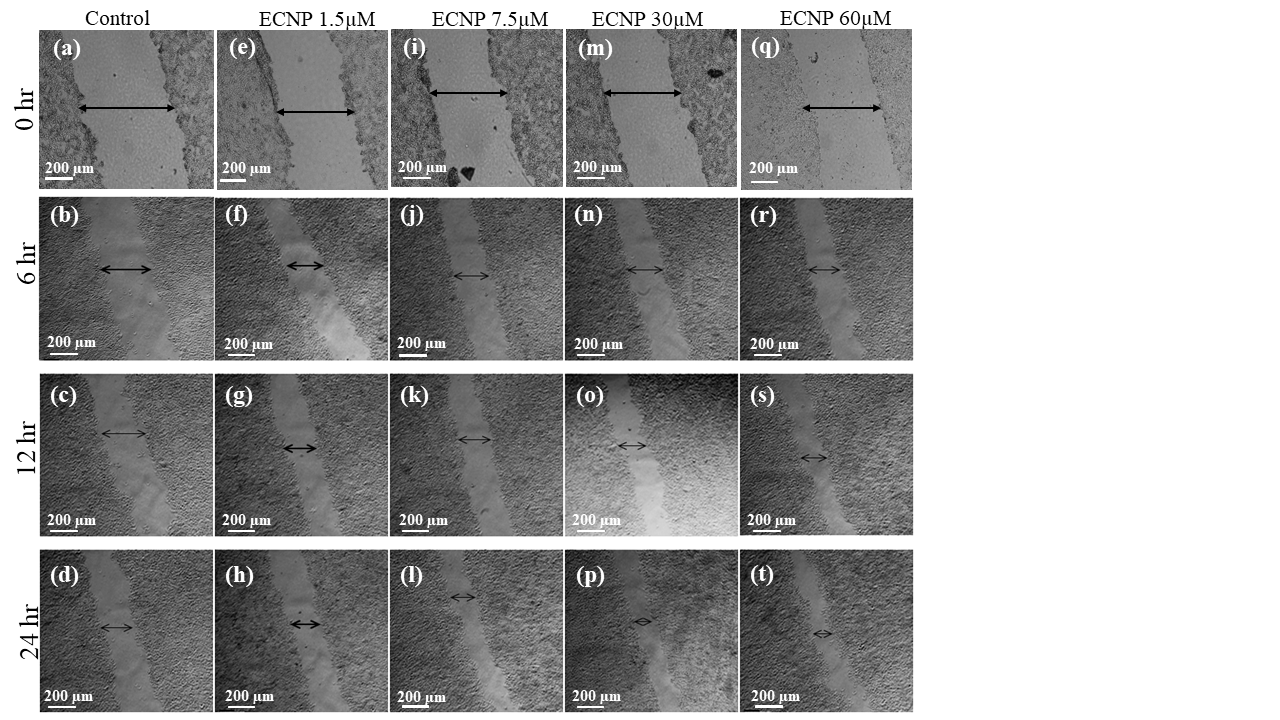


**Figure S2.** The given figure shows the effect of change in concentration of ECNP in the process of wound healing with change in time using migration assay. The cells were treated with varying concentrations of ECNP (e), (f), (g), and (h) 1.5 μM (i), (j), (k), and (l) with 7.5 μM, (m), (n), (o), and (p) with 30 μM and (q), (r), (s), and (t) with 60 μM respectively for 6, 12 and 24-hour duration to observe the cell migration.


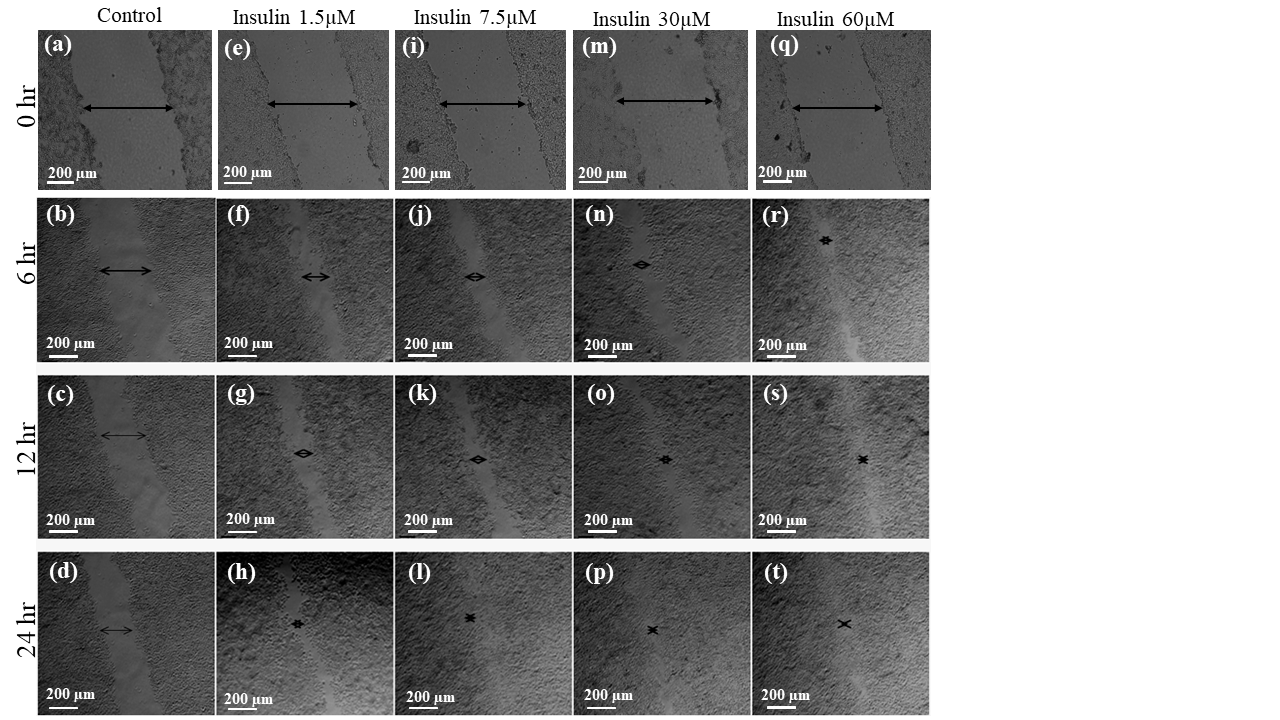


**Figure S3.** The given figure shows the effect of change in insulin concentration in the process of wound healing with change in time using migration assay. The cells were treated with varying concentrations of insulin (e), (f), (g), and (h) 1.5 μM (i), (j), (k), and (l) with 7.5 μM, (m), (n), (o), and (p) with 30 μM and (q), (r), (s), and (t) with 60 μM respectively for 6, 12 and 24-hour duration to observe the cell migration.


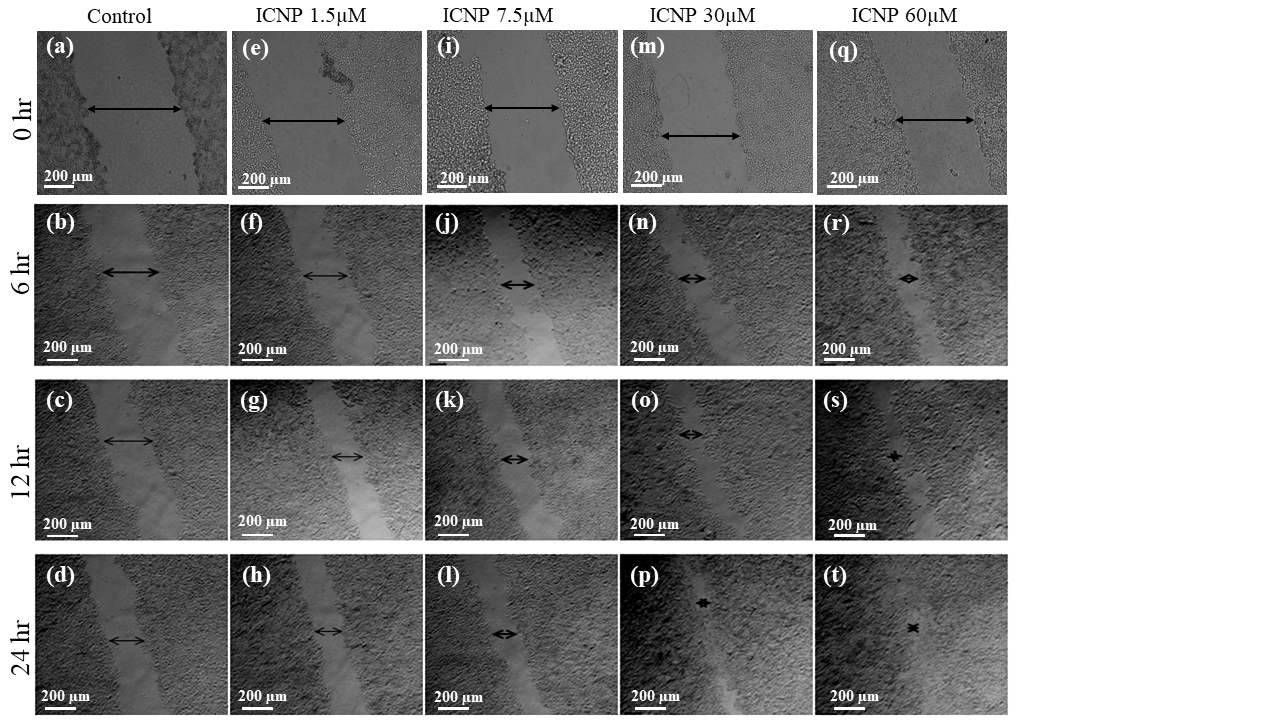


**Figure S4.** The given figure shows the effect of change in concentration of ICNP in the process of wound healing with change in time using migration assay. The cells were treated with varying concentrations of ICNP (e), (f), (g), and (h) 1.5 μM (i), (j), (k), and (l) with 7.5 μM, (m), (n), (o), and (p) with 30 μM and (q), (r), (s), and (t) with 60 μM respectively for 6, 12 and 24-hour duration to observe the cell migration.


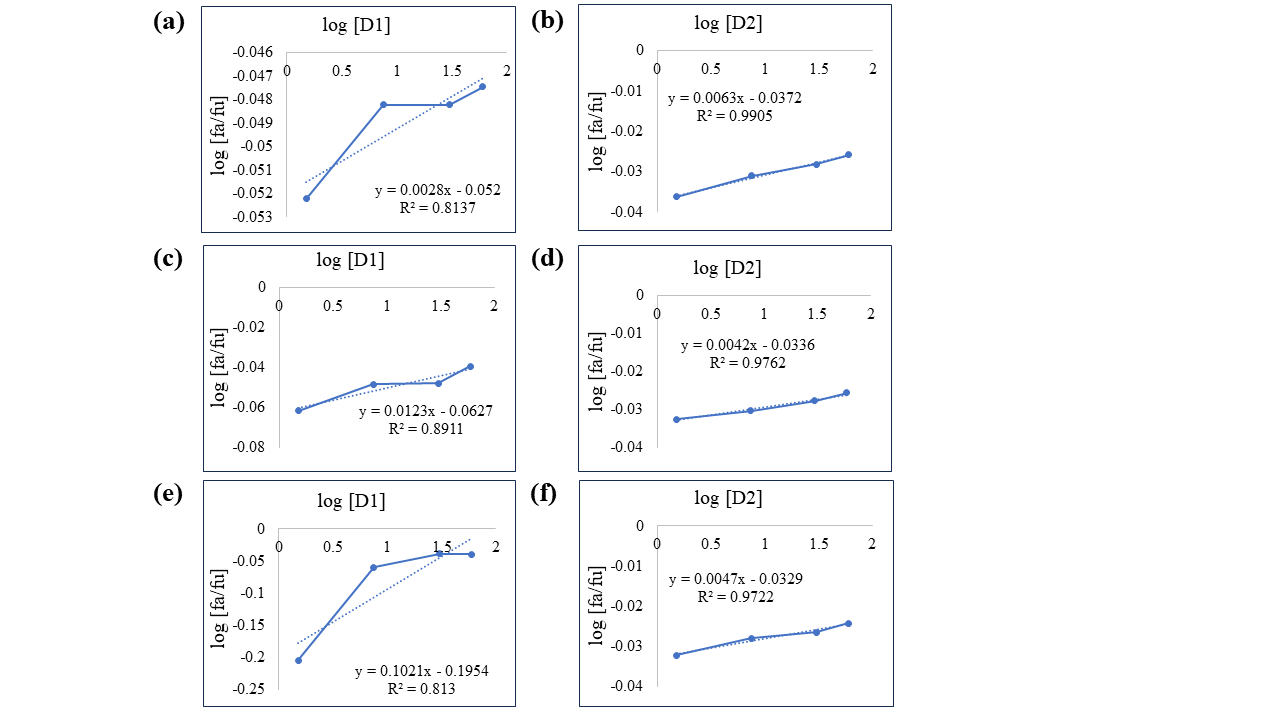


**Figure S5.** Median plots of (a) Chitosan, (b) Insulin after 6 hours, (c) Chitosan, (d) Insulin after 12 hours, and (e) Chitosan and (f) Insulin after 24 hours for finding the y-intercept and m values to calculate Dm to determine the combination index of cobalt and insulin.


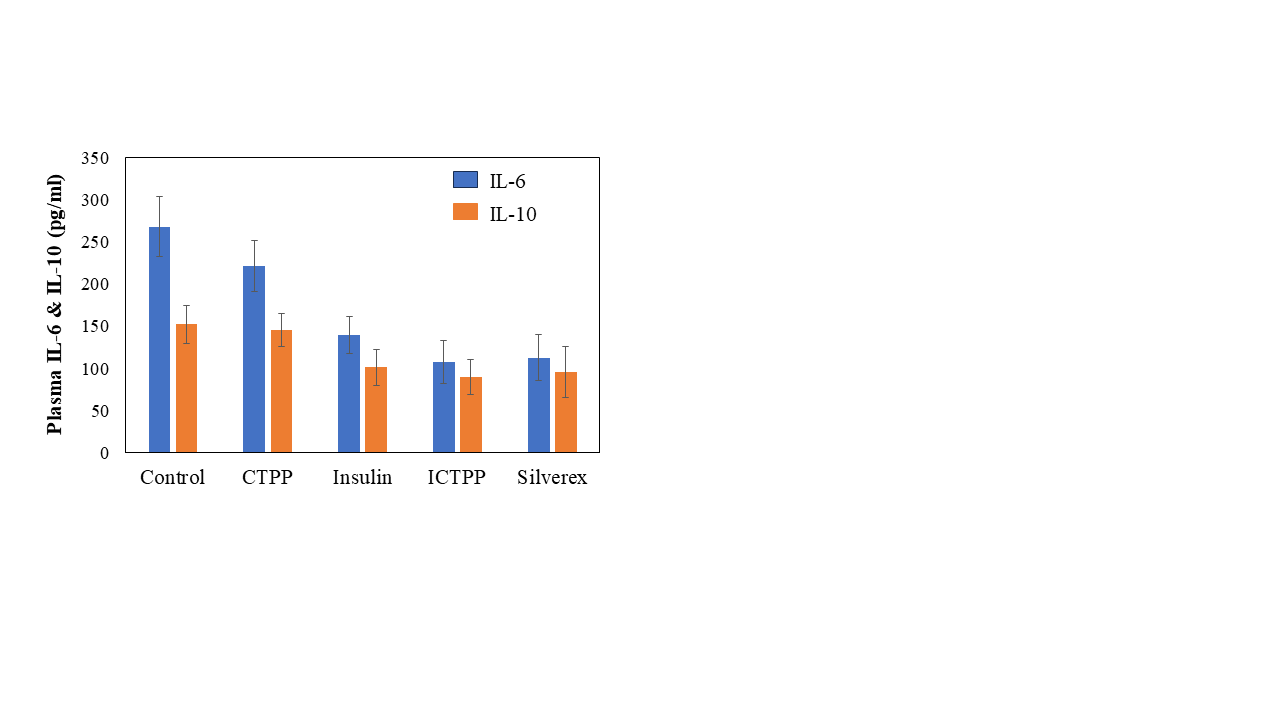


Silverex

ICNP

Insulin

ECNP

Control

**Figure S6.** The figure shows the post-burn injury plasma IL-6 and IL-10 levels collected on day 20 to analyze the inflammatory phases. Data are mean ± SEM; n=3/group.

**Table S1.** The table below depicts the zeta potential variation of ECNP and ICNP with varying pH conditions, including pH 6, 7.4, 8, and 9, respectively.

| pH | Zeta potential (mV)  ECNP | Zeta potential (mV)  ICNP |
| --- | --- | --- |
| 6 | 10.9 ± 2.74 | 28.8 ± 3.08 |
| 7.4 | 25.9 ± 3.39 | 23.2 ± 2.9 |
| 8 | 26.7 ± 2.63 | 23.4 ± 3.12 |
| 9 | 24.6 ± 3.1 | 14.7 ± 2.5 |

**Table S2.** The table shows the variation in mitochondrial reductase activity in the MTT assay for determining cellular metabolism rate using HEKa cells. The cells were treated with varying concentrations of ECNP, insulin, and ICNP, that is, 1.5 µM, 7.5 µM, 30 µM, and 60 µM respectively, for a duration of 24, 48 and 72 hours. The OD values for control after 24, 48, and 72 hours are 0.255 ± 0.020, 0.606 ± 0.036, and 0.850 ± 0.007, respectively. The data for the rest of the samples is presented below in a comparative manner and given as mean value ± SD of three independent experiments.

| **Change in mitochondrial reductase activity (OD value at 575 nm)** | | | | |
| --- | --- | --- | --- | --- |
| **Dose** | **Time** | **ECNP** | **Insulin** | **ICNP** |
| **1.5 µM** | 24 h | 0.263 ± 0.040 | 0.271 ± 0.017 | 0.287 ± 0.033 |
|  | 48 h | 0.606 ± 0.036 | 0.634 ± 0.062 | 0.741 ± 0.050 |
|  | 72 h | 0.869 ± 0.066 | 0.940 ± 0.056 | 1.156 ± 0.081 |
| **7.5 µM** | 24 h | 0.269 ± 0.037 | 0.286 ± 0.037 | 0.336 ± 0.097 |
|  | 48 h | 0.624 ± 0.048 | 0.659 ± 0.051 | 0.782 ± 0.043 |
|  | 72 h | 0.872 ± 0.141 | 1.201 ± 0.098 | 1.371 ± 0.084 |
| **30 µM** | 24 h | 0.279 ± 0.010 | 0.288 ± 0.021 | 0.348 ± 0.026 |
|  | 48 h | 0.634 ± 0.062 | 0.745 ± 0.048 | 0.847 ± 0.077 |
|  | 72 h | 1.0167 ± 0.125 | 1.407 ± 0.154 | 1.46 ± 0.026 |
| **60 µM** | 24 h | 0.284 ± 0.012 | 0.331 ± 0.067 | 0.367 ± 0.037 |
|  | 48 h | 0.678 ± 0.077 | 0.741 ± 0.082 | 0.875 ± 0.058 |
|  | 72 h | 0.957 ± 0.059 | 1.439 ± 0.067 | 1.575 ± 0.121 |

**Table S3.** It shows the p values calculated for % variation in wound diameter after treatment with varying concentrations of ECNP, insulin, and ICNP (1.5, 7.5, 30, and 60 µM) after 6h, 12 h, and 24h, respectively. The statistical significance of data is considered when p < 0.05.

| **P value for checking the statistical significance of data for scratch assay** | | | | |
| --- | --- | --- | --- | --- |
| **Time** | **Concentration** | **ECNP** | **Insulin** | **ICNP** |
| **6 h** | 1.5 µM | 0.002317 | 5.68E-05 | 0.013705 |
|  | 7.5 µM | 9.94E-05 | 7.9E-05 | 9.53E-05 |
|  | 30 µM | 8.97E-05 | 1.55E-05 | 6.51E-05 |
|  | 60 µM | 8.5E-05 | 7.11E-06 | 4.13E-05 |
| **12 h** | 1.5 µM | 0.000624 | 4.03E-05 | 0.000861 |
|  | 7.5 µM | 0.001332 | 3.61E-05 | 0.000219 |
|  | 30 µM | 0.000174 | 2.09E-05 | 6.11E-05 |
|  | 60 µM | 0.000165 | 1.86E-05 | 2.88E-05 |
| **24 h** | 1.5 µM | 0.321863 | 0.00015 | 0.019979 |
|  | 7.5 µM | 0.015416 | 4.51E-05 | 0.001427 |
|  | 30 µM | 0.000193 | 1.05E-05 | 0.000112 |
|  | 60 µM | 6.91E-05 | 9.11E-06 | 0.000156 |

**Table S4.** The Combination Index (CI) values for the cell viability for varying combinations of chitosan with insulin protein were calculated to check if the two drugs are synergistic or antagonistic, and the values come out to be less than 1, indicating the synergistic effect of drugs.

| **Combination Index (CI)** | | | |
| --- | --- | --- | --- |
| The concentration of Chitosan and Insulin | (Dx)1 (Chitosan) = Dm [fa/fu]^1/m^ | (Dx)2 (Insulin) = Dm [fa/fu]^1/m^ | CI = (D)1/(Dx)1 + (D)2/(Dx)2 |
| 6 hours | | | |
| 1.5 µM | 536974719 | 6906.95751 | 0.000217 |
| 7.5 µM | 124279920 | 3473.38471 | 0.002159 |
| 30 µM | 124279920 | 2445.95753 | 0.012265 |
| 60 µM | 95113804.3 | 1832.71399 | 0.032739 |
| 12 hours | | | |
| 1.5 µM | 6081.89 | 31223.2 | 0.00029 |
| 7.5 µM | 1853.28 | 20645 | 0.00441 |
| 30 µM | 1786.88 | 12640 | 0.01916 |
| 60 µM | 896.3 | 8703.2 | 0.07384 |
| 24 hours | | | |
| 1.5 µM | 1918.72 | 2159.44 | 0.00148 |
| 7.5 µM | 588.579 | 1368.32 | 0.01822 |
| 30 µM | 304.722 | 1167.8 | 0.12414 |
| 60 µM | 317.224 | 937.648 | 0.25313 |

**Table S5.** It shows the p values calculated for checking the statistical significance of data for IL-6, IL-10 and Nrf-2 % variation in wound diameter after treatment with ECNP, insulin, and ICNP (60 µM) respectively. The statistical significance of data is considered when p < 0.05.

| **P value for checking the statistical significance of data for IL-6, IL-10 and Nrf-2** | | | | |
| --- | --- | --- | --- | --- |
|  | **ECNP** | **Insulin** | **ICNP** | **Silverex** |
| **IL-6** | 0.0678 | 0.0481 | 0.000612 | 0.00457 |
| **IL-10** | 0.0751 | 0.0547 | 0.0081 | 0.0518 |
| **Nrf-2** | 0.0524 | 0.00042 | 0.000181 | 0.000524 |
